# Vistas in Non-Small Cell Lung Cancer (NSCLC) Treatment: of Kinome and Signaling Networks

**DOI:** 10.7150/ijbs.83574

**Published:** 2023-04-01

**Authors:** Dimitrios J. Stravopodis, Kostas A. Papavassiliou, Athanasios G. Papavassiliou

**Affiliations:** 1 Section of Cell Biology and Biophysics, Department of Biology, National and Kapodistrian University of Athens, 15701 Athens, Greece.; 2 First University Department of Respiratory Medicine, 'Sotiria' Hospital, Medical School, National and Kapodistrian University of Athens, 11527 Athens, Greece.; 3 Department of Biological Chemistry, Medical School, National and Kapodistrian University of Athens, Athens 11527, Greece.

**Keywords:** Non-small cell lung cancer (NSCLC), Human kinome activity, Serine/threonine protein kinases, Tyrosine protein kinases, Signal transduction networks, Therapeutic strategies

## Abstract

Non-small cell lung cancer (NSCLC) is the prevailing lung cancer type, accounting for ~85% of all lung cancer cases. Despite their initial promise, current chemotherapeutic protocols are reaching their limits. This necessitates the prompt discovery of new molecular drivers and the development of novel regimens for advanced NSCLC. Herein, we pose that there is a need to systematically profile the human kinome activity of NSCLC. Using available state-of-the-art technologies, a wide gamut of kinase activities can be simultaneously mapped and quantified specifically in the primary or metastatic cancer states, with oncogenic kinase functions being likely linked to mutation signatures and malignant features of NSCLC. New chemical compound libraries can then be screened for kinase inhibitory properties in preclinical model systems, with presumptive induction of programmed cell-death subroutines and signaling-disintegration routes serving as major outputs of novel inhibitor tumor-suppressor potentials.

## Challenges in non-small cell lung cancer (NSCLC) therapeutics

Lung cancer has been recently reported as the second most frequent human malignancy and the major cause of cancer-related mortality worldwide 1. It is classified into two main types, namely, non-small cell lung cancer (NSCLC) and small cell lung cancer (SCLC), with NSCLC accounting for approximately 85% of all cases 2. NSCLC is further categorized into subtypes including adenocarcinoma, squamous cell carcinoma, large cell carcinoma and other variants, with lung adenocarcinoma and squamous cell carcinoma representing the largest NSCLC subgroups.

Oncogenic mutations in *Kirsten RAt Sarcoma* (*KRAS*) and *Epidermal Growth Factor Receptor* (*EGFR*) genetic loci are frequently detected in NSCLC cases. Early-stage lung adenocarcinoma is associated with 29.1% and 14.2% of *KRAS* and *EGFR* genomic alterations, respectively, while metastatic adenocarcinoma is related to 29.9% and 30.3% of the respective oncogenic aberrations 3. A significant spectrum expansion of the targetable and druggable genomic alterations in NSCLC was achieved by the discovery of *Anaplastic Lymphoma Kinase* (*ALK*) gene rearrangements (fusions; 0.8% in early and 4.4% in the metastatic disease), together with co-occurring pathogenic drivers that include, among others, the mutated Fibroblast Growth Factor Receptors 1/2 (FGFRs1/2), neuroblastoma Ras (N-Ras), REarranged during Transfection (RET) and ROS proto-oncogene 1 (ROS1) cell signaling protein components 3.

Targeted, as opposed to cytotoxic (e.g., cisplatin-based), chemotherapy (and immunotherapy) is considered as a relatively selective and rather safe approach, capable of markedly improving NSCLC patient prognosis. Next-generation inhibitors targeting oncogenic activities of EGFR or ALK molecular drivers in advanced NSCLCs harboring gain-of-function respective mutations, have shown beneficial effects in median survival 4. Nevertheless, *KRAS* mutations are notably common in lung adenocarcinoma, with *KRAS*^G12C^ number exceeding any other number of targetable mutations including *EGFR* and *ALK* 5, thereby entailing the prompt development of drugs targeting mutant K-Ras. Notwithstanding the long line of failed attempts, novel compounds have been recently discovered that inhibit *KRAS*^G12C^ oncogenic activity 5, 6. However, their therapeutic capacity may be severely compromised due to intrinsic/acquired resistance mechanisms 4, 7.

## NSCLC kinomics

Signaling adaptation-triggered limitations of *KRAS*^G12C^ inhibition efficacy can be overcome by the synergistic action of PhosphoInositide 3-Kinase (PI3K) inhibitors 8, thus revealing the pivotal roles of active/ated kinases in tumor initiation, progression, metastasis and resistance to therapy. In contrast, NSCLC that lacks targetable mutations, combination chemotherapy (e.g., cisplatin and gemcitabine) schemes are the main options, with immunotherapy (e.g., agents (peptides, small-molecule chemical compounds and antibodies) targeting the Programmed cell Death protein-1 (PD-1)/Programmed cell Death-Ligand 1 (PD-L1) axis) also contributing to the clinical treatment of advanced disease 4. Interestingly, preclinical studies suggest the therapeutic potential of EGFR Tyrosine Kinase Inhibitors (TKIs) in combination with immunotherapy for NSCLC treatment 9, 10. Such data have paved the way for clinical trials evaluating the combination of kinase inhibitors (e.g. EGFR or JAnus Kinase (JAK) inhibitors) with immune checkpoint inhibitors in NSCLC (ClinicalTrials.gov Identifiers: NCT02630186, NCT03425006). Altogether, it seems that novel kinases and their specific inhibitors need to be identified to efficiently target NSCLC with already known or new oncogenic drivers.

**Figure 1:**
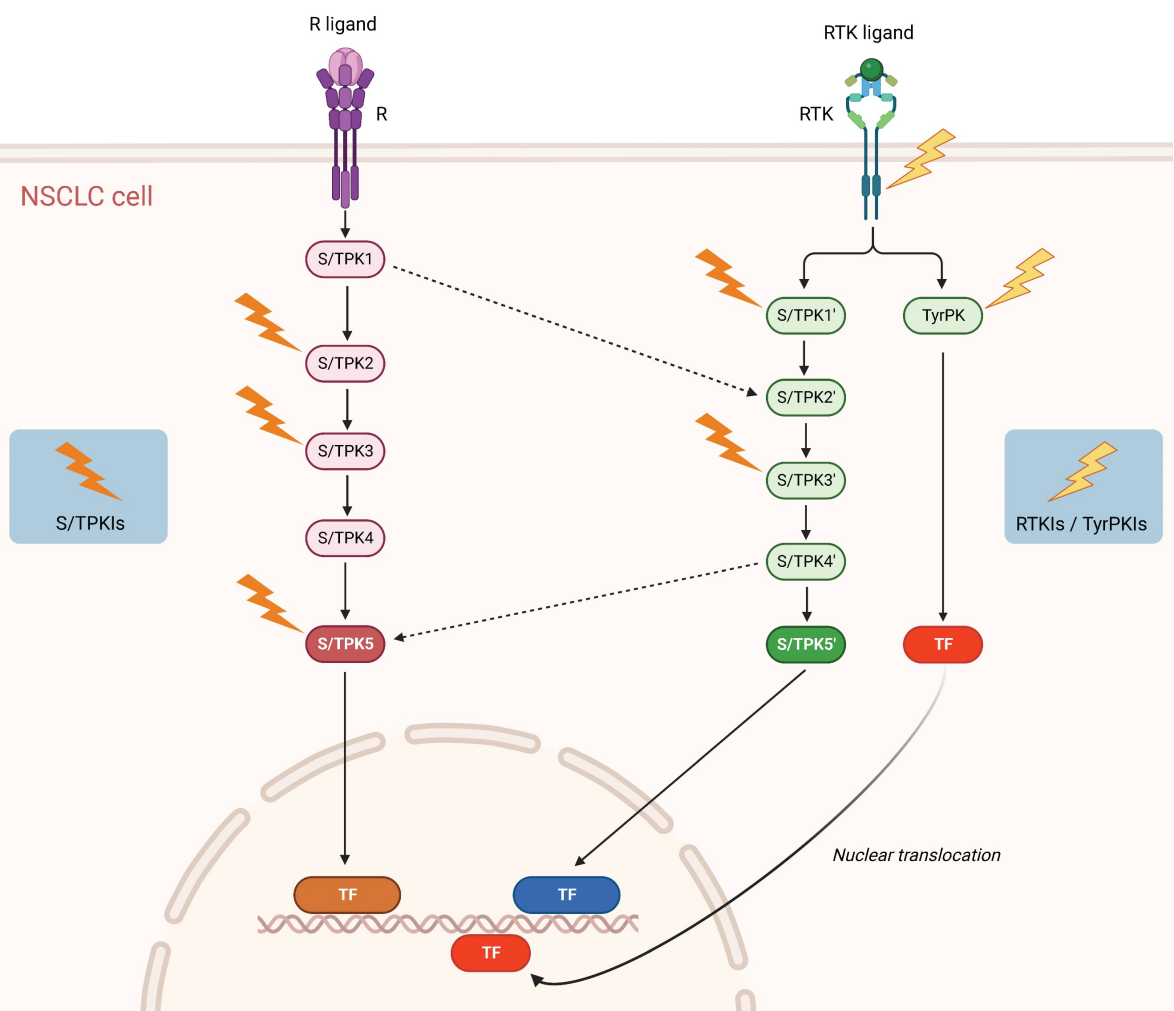
Schematic representation of signaling pathways and potential targets in NSCLC. Solid arrows denote activating protein (kinase) phosphorylation events and dashed arrows kinase crosstalk between two different signal transduction cascades. Lightning symbols indicate potential kinase targeting by specific inhibitors. R: Receptor (e.g. Tumor Necrosis Factor Receptor 1 (TNFR1)); RTK: Receptor Tyrosine Kinase (e.g. Epidermal Growth Factor Receptor (EGFR), Mesenchymal-Epithelial Transition factor (MET)); S/TPK: Serine/Threonine Protein Kinase; TyrPK: Tyrosine Protein Kinase; TF: NSCLC-implicated Transcription Factor (e.g. Activator Protein-1 (AP-1), Nuclear Factor-kappa B (NF-κB), Signal Transducer and Activator of Transcription 3 (STAT3)); S/TPKIs: Serine/Threonine Protein Kinase Inhibitors; RTKIs: Receptor Tyrosine Kinase Inhibitors; TyrPKIs: Tyrosine Protein Kinase Inhibitors. This figure was created based on the tools provided by Biorender.com (accessed on 14/03/2023).

Accordingly, the NSCLC-specific activities (and not simply expression levels and/or mutational states) of multiple protein kinases should be profiled, in comparison to healthy tissues, both in the primary and metastatic areas. Utilizing validated high-throughput protein kinase activity profiling technologies, serine/threonine and tyrosine protein kinase species displaying the most important functional differences between primary tumor versus healthy samples and primary versus metastatic tumor specimens can be assembled in signal transduction networks, via sophisticated bioinformatic platforms. In that vein, the implementation of Artificial Intelligence (AI) approaches in high-throughput anatomopathological and histological analyses, including immunohistochemical studies, is becoming more and more relevant. Consequently, a new multiple active-kinase (active-kinome) signature can be developed, likely opening new therapeutic avenues for the disease (**Figure 1**).

To verify this active-kinome chart, NSCLC cell lines obtained from either primary or metastatic (lymph nodes) tumors, together with normal lung epithelial cells (serving as control), can be targeted with commercially available or chemically-synthesized specific kinase inhibitors, in mono-therapy and cocktail-therapy schemes. Their apoptotic, autophagic and necroptotic/necrophagic cell-death responses can thus be studied, via employment of 3-[4,5-dimethylthiazol-2-yl]-2,5 diphenyl tetrazolium bromide (MTT), Western immunoblotting, Reverse Transcription-quantitative real-time Polymerase Chain Reaction (RT-qPCR), Fluorescence-Activated Cell Sorting (FACS) and ImmunoFluorescence (IF) assays. Additionally, Severe Combined Immune Deficient (SCID) mice-tumor xenografts can be treated *in vivo* with the most efficient anti-oncogenic kinase inhibitors (single- or multiple-compound regimens), and tumor size and growth rate be analyzed using both conventional biometrics and luciferase-based *in vivo* imaging tools.

This druggable map of NSCLC-specific active kinome offers a valuable toolbox to proficiently combat and overcome the molecular diversity and acquired resistance frequently observed during therapy of advanced and metastatic NSCLC. The application of AI tools will be of outmost importance in the analysis of this kinome network, allowing prediction of drug resistance mechanisms and identification of potential synthetic lethalities.

## Concluding remarks - Outlook

Given the cellular heterogeneity, heavy mutation load and frequent resistance to therapy of NSCLC, new “omics” strategies must be promptly developed to successfully cope with knotty and tangly molecular networks that govern NSCLC pathobiology. Novel mutation signatures, mechanistic/functional biomarkers and targeted drug schemes must be discovered in order to significantly reduce the mortality of advanced, metastatic and chemoresistant NSCLC. To this end, mutation burden, active-kinome profile and kinase inhibition are expected to serve as a powerful and precious toolbox for the successful management of NSCLCs in the clinical setting. This new prospect of kinome activity-dependent tumor drugging and therapeutics will likely result in multiple benefits, including lower cost of health-care system, reduced hospitalization and sick-leave time, shorter recovery time after chemotherapy, lower systemic toxicity and decreased regimen price. NSCLC subtype-dependent mutation maps, together with tumor-specific kinome activities and their targeted drugging with new generations of chemically-synthesized inhibitors, will most probably improve the prognosis of advanced, metastatic and chemoresistant NSCLC patients.

In summary, the aforementioned treatment plan holds strong promise for the efficient, safe and economically abating therapy of NSCLC patients.
